# Characterization of *Escherichia coli* Isolated from Sows Presenting Purulent Vulvar Discharge

**DOI:** 10.3390/microorganisms12010123

**Published:** 2024-01-08

**Authors:** André P. Poor, Luisa Z. Moreno, Matheus S. Monteiro, Carlos E. C. Matajira, Maurício C. Dutra, Diego F. Leal, Ana Paula S. Silva, Vasco T. M. Gomes, Ivan O. de Souza, Kawany M. Araújo, Maria Inês Z. Sato, Andrea M. Moreno

**Affiliations:** 1 Department of Preventive Veterinary Medicine and Animal Health, School of Veterinary Medicine and Animal Science, University of São Paulo, Av. Prof. Dr. Orlando Marques de Paiva 87, São Paulo 05508-270, SP, Brazil; andrepegoraro21@gmail.com (A.P.P.); luzanolli@gmail.com (L.Z.M.); matheus.salibamonteiro@gmail.com (M.S.M.); k.rlos89.cabrera@gmail.com (C.E.C.M.); maucdutra@hotmail.com (M.C.D.); anapaula_silva2006@yahoo.com.br (A.P.S.S.); vaskotulio@yahoo.com.br (V.T.M.G.); ivanochin@usp.br (I.O.d.S.); kawmaraujo@gmail.com (K.M.A.); 2 Facultad de Ciencias Básicas, Universidad Santiago de Cali, Calle 5 #62-00, Cali 760035, Colombia; 3 Department of Animal Production and Nutrition, School of Veterinary Medicine and Animal Science, University of São Paulo, Pirassununga 13635-900, SP, Brazil; diegoleal6446@hotmail.com; 4 Environmental Company of the State of São Paulo (CETESB), Av. Prof. Frederico Hermann Júnior 345, São Paulo 05459-900, SP, Brazil; misato@sp.gov.br

**Keywords:** urogenital infections, sows, *Escherichia coli* virulence, antimicrobial resistance

## Abstract

Purulent vulvar discharge is a clinical sign of genitourinary tract infections, which are a significant concern in swine facilities, leading to sow culling and mortality. *Escherichia coli* is one of the main agents involved in these diseases. This study aimed to characterize the virulence and antimicrobial resistance profiles as well as the phylotype of *Escherichia coli* strains isolated from sows with purulent vulvar discharge. The results showed that at least 2 of the 29 tested virulence genes related to extraintestinal pathogenic *E. coli* were present in all strains tested. The most frequent gene was *iutA*, present in all strains, followed by the genes *iucD*, *csgA*, *iss2*, and *irp2*. Associations between iron uptake genes, genes related to adhesion, attachment, and serum resistance, as well as genes related to toxin release and bacteriocin, were frequent. The most prevalent phylotype was B1 (40.0%), followed by A (18.5%), D (11.9%), C (9.6%), B2 (7.4%), E (4.4%), F (1.5%), and Clade I (0.7%), with B2 being related to highly virulent traits. The strains presented elevated resistance to antimicrobials such as ciprofloxacin, streptomycin, cephalexin, florfenicol, and ampicillin. More than 90% of the strains were identified as multidrug-resistant, indicating the selection that is induced by the high use of antimicrobials in swine farming.

## 1. Introduction

*Escherichia coli* is a commensal agent that can acquire certain virulence attributes and become associated with a wide range of diseases in domestic animals. Based on the presence of these distinct attributes and the location of infection, these strains have been classified into pathotypes [1]. These strains can impair production due to mortality and morbidity and also incur direct financial losses from treatment costs. Furthermore, they may also compromise food quality, representing a one health issue [2].

In swine, *E. coli* is one of the primary agents isolated in cases of genitourinary infection and vulvar discharge [3,4]. Therefore, several studies have related the virulence factors associated with infections affecting sites other than the intestine, such as the reproductive tract, designating this pathotype as ExPEC (extraintestinal pathogenic *Escherichia coli*). Morbidity and mortality in human cases caused by ExPEC are increasing globally [5], and the importance of swine ExPEC is also being recognized [6,7,8]. However, few studies have comprehensively characterized the virulence factors of *E. coli* isolated from the genitourinary tract of female pigs. In Brazil, studies that characterized the virulence factors of uropathogenic *E. coli* found that genes for P fimbriae, type 1 fimbriae, and iron acquisition systems are among the most prevalent [9,10].

The phylogenetic characterization proposed by Clermont et al. [11,12] has been applied to several *E. coli* isolated from animal hosts and human clinical cases. In human virulent strains, the phylogenetic groups B2 and D are more frequent, whereas group A is more related to commensal strains [2].

In this study, *E. coli* strains isolated from sows with purulent vulvar discharge were evaluated for the presence of ExPEC virulence genes, had their phylogenetic group determined, and were assessed for their resistance profile to antimicrobials commonly used in pig farming.

## 2. Materials and Methods

### 2.1. Ethics Committee Approval

The Animal Use Ethics Committee (CEUA) of the School of Veterinary Medicine and Animal Science, University of São Paulo, approved this study under the CEUA Process Number 1875170317.

### 2.2. Animal Sampling

Samples were collected from the deep region of the vaginal canal of animals with vulvar discharge from four commercial breeding systems situated in the Brazilian states of São Paulo, Paraná, Minas Gerais, and Mato Grosso, which had a recent history of increased rates of metritis and abortions. A total of 107 hybrid commercial females (Landrace × Large White) were collected, with parity ranging between 1 and 10, with an average of 3.6. All farms perform artificial insemination of sows. The sample collection was carried out using a sterile and disposable vaginal speculum; sows were evaluated in the pregnancy or lactation periods, usually three days after farrowing. Herds were initially screened based on the presence of purulent material on the females’ enclosures. To minimize the risk of external contamination, two veterinarians conducted the sampling—one was responsible for exposing the vaginal canal while the other carried out the speculum and swab handling.

Only animals presenting purulent secretions in the deep region of the vaginal canal were sampled, which ruled out the possibility of vulvar lesions or vaginitis. In addition, only exclusively purulent secretions, strongly suggestive of infectious processes, were collected, while normal-appearing secretions and lochia were not included in the sampling. Similarly, sows that had recently urinated were not sampled. The swabs were placed in Stuart’s transport medium and kept refrigerated at 4 °C until they arrived at the laboratory for further processing.

### 2.3. Bacterial Isolation and Identification

For *E. coli* isolation, swabs were streaked on MacConkey agar and Chromagar™ Orientation (Difco, Sparks, MD, USA) and incubated at 37 °C for 24 h under aerobic conditions. Suspected bacterial colonies were sub-cultured in 3.0 mL of Brain Heart Infusion (BHI) broth (Difco, Sparks, MD, USA), and aliquots were taken from this culture for DNA extraction and strain identification using Matrix Associated Laser Desorption-Ionization—Time of Flight (MALDI-TOF) mass spectrometry.

For MALDI-TOF MS, ribosomal protein extraction was performed as described by Hijazin et al. [13]. The Microflex™ mass spectrophotometer (Bruker Daltonics, Inc., Billerica, MA, USA) from the Environmental Company of the State of São Paulo (CETESB) was used. The protein spectra were captured by the FlexControl™ (Bruker Daltonics, Inc., Billerica, MA, USA) using the MTB_autoX method. The spectrophotometer was externally calibrated using the Bacterial Test Standard (BTS—Bruker Daltonics, Inc., Billerica, MA, USA). The microbial identification was performed by BioTyper™ 3.0 (Bruker Daltonics, Inc., Billerica, MA, USA) using the following manufacturer criteria: species were assigned log score values ≥ 2.0, while scores between ≥ 1.7 and <2.0 determined only genus identification.

### 2.4. DNA Extraction and Polymerase Chain Reactions (PCRs) for Phylogenetic Characterization and ExPEC Virulence Gene Screening

The bacterial DNA was extracted according to Boom et al. [14] protocol and maintained at −20 °C until processing. The *E. coli* phylogenetic characterization was performed as described by Clermont et al. [11,12], enabling the strain classification into eight phylogenetic groups (A, B1, B2, C, D, E, F, and *Escherichia* crypt Clade I). A total of 29 genes were evaluated that encode virulence factors associated with ExPEC, as described in different studies [7,15,16,17,18,19,20,21,22,23,24,25,26,27]. The primers for each gene and the references are described in Table S1.

The PCRs (50 µL) used 5 µL of genomic DNA, ultrapure water, 10X PCR buffer, 1.5 mM MgCl2, 200 µM of dNTPs, 20 pmol of each primer, and 1 U of HOT FIREPol DNA-polymerase (Solis BioDyne, Tartu, Estonia). The PCR cycles followed the respective protocol reference. Amplicons were detected by agarose gel electrophoresis (1.5%) stained with BlueGreen^®^ (LGC Biotecnologia, Cotia, SP, Brazil). Images were captured under UV illumination by the Gel Doc XR system (Bio-Rad Laboratories, Hercules, CA, USA), and the 100 bp DNA Ladder molecular weight marker (New England BioLabs Inc., Ipswich, MA, USA) was used for further band analysis.

### 2.5. Antimicrobial Resistance Characterization

The characterization of the antimicrobial resistance profile was performed by a broth microdilution test according to the standards defined in the VET01 document [28]. For the inoculum preparation, strains were cultured in BHI broth (Difco, Sparks, MD, USA) and incubated at 37 °C for 24 h. The turbidity of the culture was adjusted with sterile saline solution (0.9%) to obtain an optical density comparable to that of the 0.5 McFarland standard solution and confirmed by spectrophotometer (0.150 to 600 nm). This adjusted bacterial suspension had approximately 1.0 to 2.0 × 10^8^ CFU/mL, which was further diluted by 1:1000 in Mueller–Hinton II broth (Difco, Sparks, MD, USA) to obtain a final concentration of approximately 5.0 × 10^5^ CFU/mL. Subsequently, 50 µL of this final suspension was distributed in each well of the microplate, which was sealed, incubated at 37 °C, and assessed within 18 h of incubation. The minimum inhibitory concentration (MIC) of each antimicrobial was assessed visually and was established as the lowest concentration of antimicrobial without button formation.

*Staphylococcus aureus* ATCC 29213 and *Escherichia coli* 25922 strains were used as quality controls. The MIC_50_ and MIC_90_ were determined as described by Schwarz et al. [29].

### 2.6. Statistical Analysis

The statistical analyses were performed with R-Studio software (version 2023.12.0) [30]. The Fisher’s exact test (two-tailed) and the Chi-square test were performed using a statistical significance level of *p* ≤ 0.05, adjusted with Bonferroni correction for multiple tests.

The upset plots were generated for the representation of the virulence profiles of *Escherichia coli* strains using the Complex Heatmap package [31]. Correspondence analysis and heatmaps were performed using factoextra, FactoMineR, and ggplot2 [32,33,34]. Comparisons were performed between virulence gene groups and resistance groups. The gene groups were divided according to the number of virulence genes present in the strains: between two and four genes, between five and seven genes, between eight and ten genes, and more than ten genes. The resistance groups were divided as resistance to one to three antimicrobials, four to six antimicrobials, seven to ten antimicrobials, and resistance to more than ten of the tested antimicrobials. Phylotypes were also compared with virulence groups.

## 3. Results

### 3.1. Phylogenetic Characterization

A total of 135 *E. coli* strains were selected from 107 positive animals and identified using MALDI-TOF MS, which were submitted to phylogenetic characterization and further screening for virulence genes related to extraintestinal infection.

From the 135 *E. coli* strains obtained, it was possible to classify most strains (94.1%) within the eight phylotypes; only eight strains (5.9%) could not be classified. Most of the strains were classified as belonging to the B1 group (40%), followed by group A (18.5%). The rest of the strains were divided into groups D (11.9%), C (9.6%), B2 (7.4%), E (4.4%), F (1.5%), and Clade I (0.7%). Phylotype B1 exhibited a higher prevalence when compared to all other phylotypes (*p* < 0.05, Chi-square test), except when compared to phylotype A.

### 3.2. ExPEC-Associated Virulence Gene Screening

**Figure 1:**
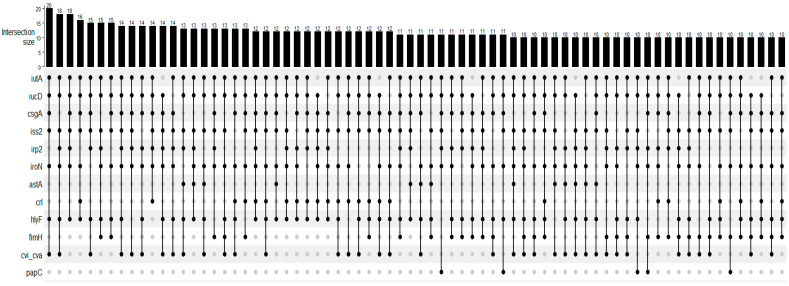
UpSet plot for the number of occurrences of main virulence profiles of *Escherichia coli* strains isolated from females with purulent vulvar discharge (*n* = 135). The numbers above the bars show the number of strains in which genes, indicated by the filled dots, were detected in association.

The tested virulence genes and their frequencies are presented in Table 1. The most frequent gene was *iutA*, present in all strains, followed by *iucD*, *csgA*, *iss2*, and *irp2* genes. The *fyuA*, *afa*, *afaBC*, *papE*, and kpsMTIII genes were not detected. At least 2 of the 29 tested genes were detected in all tested strains, and a maximum of 14 genes were detected in one strain. The median was seven virulence genes harbored by the strains. Figure 1 presents the UpSet plot for the virulence gene association detected in the strains. Iron uptake (*iutA*, *iucD*, *irp2*, *iroN)*, genes related to adhesion and attachment (*csgA*, *fimH*, *papC*), and genes related to serum resistance, toxin release, and bacteriocin (*iss2*, *hlyF*, *cvi/cva*) were the most prevalent in these associations.

**Figure 2:**
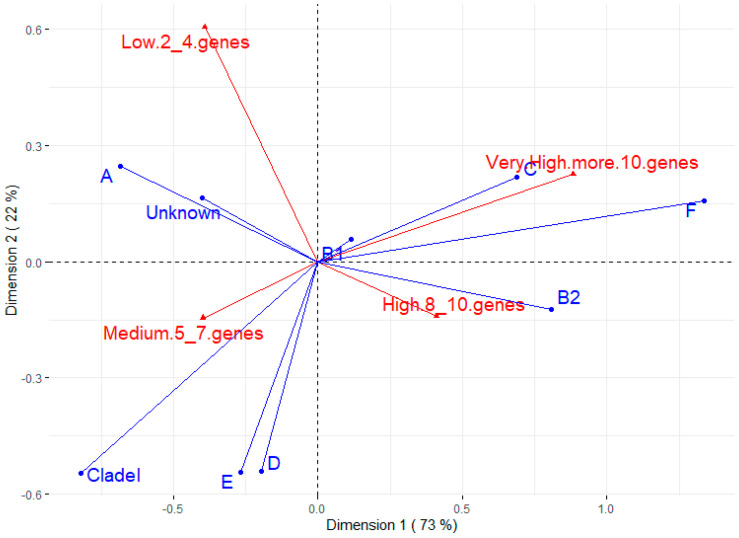
Correspondence analysis for phylotype and number of virulence genes. The groups for virulence genes were divided into low (strains having 2 to 4 virulence genes), medium (5 to 7 genes), high (8 to 10 genes), and very high (more than 10 virulence genes).

Figure 2 shows the correspondence analysis between phylotypes and the number of virulence genes. The most prevalent phylotype (B1) does not appear to be associated with any virulence trait. On the other hand, B2 seems to have a strong positive association with strains of high virulence (between eight and ten virulence genes), which is corroborated by the Chi-square test between B2 and other phylotypes for some virulence factors (Supplementary Table S2). The C and F phylotypes also seem to have a positive relationship with strains of very high virulence (more than 10 genes). Phylotype A and untyped strains are related to lower virulence, while phylotypes D, E, and Clade I seem to be associated with medium virulence (five to seven genes).

### 3.3. Antimicrobial Resistance Profile

**Figure 3:**
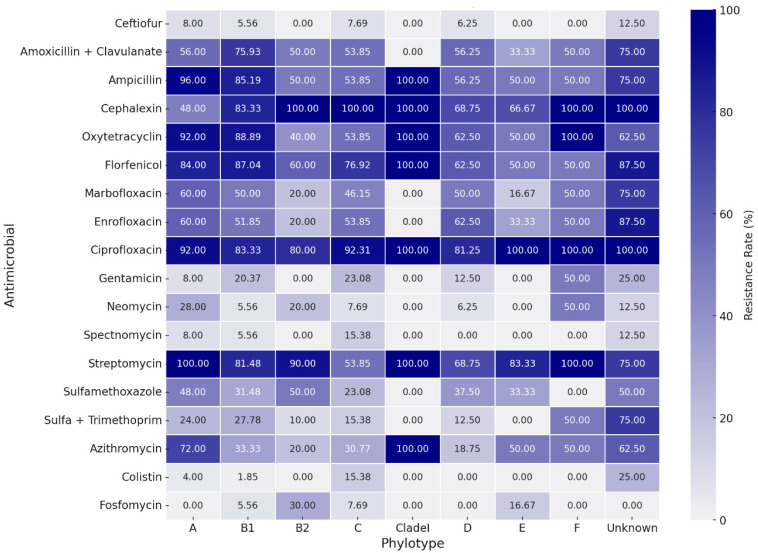
Heatmap for antimicrobial resistance rates between phylotypes.

**Figure 4:**
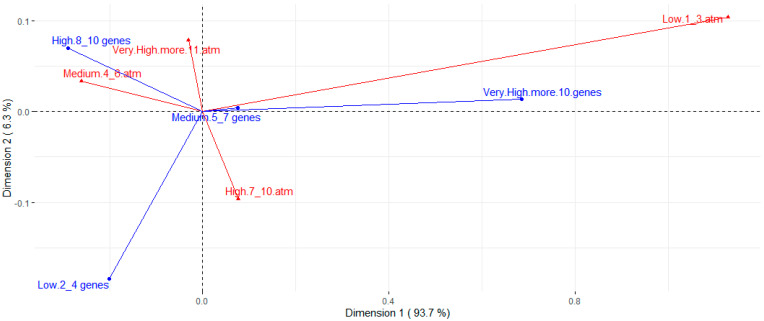
Correspondence analysis for number of virulence genes (blue arrows) and resistance to different antimicrobials (red arrows). Resistance groups were divided into low (strains are resistant to 1 to 3 of the antimicrobials tested), medium (resistant to 4 to 6 antimicrobials), high (resistant to 7 to 10 antimicrobials), and very high (resistant to 11 or more antimicrobials).

Table 2 presents the assessed concentrations, cut-off points, and resistance rates for each tested antimicrobial. The strains presented a high resistance rate to several classes of antimicrobials. Higher resistance rates, around 80%, were observed for ciprofloxacin, streptomycin, cephalexin, florfenicol, ampicillin, and oxytetracycline. A high resistance rate was also observed for the amoxicillin-clavulanic acid association (63.0%). Except for streptomycin, the aminoglycoside class (gentamicin, neomycin, and spectinomycin) showed good action against *E. coli* strains, along with colistin, fosfomycin, trimethoprim-sulfamethoxazole, and ceftiofur. The strains showed intermediate resistance rates to enrofloxacin, marbofloxacin, sulfamethoxazole, and azithromycin, ranging from 36.3% to 53.3% of resistance. There was a wide distribution of resistance rates among phylogroups (Figure 3). However, higher resistance rates are noted for groups A and B1 when compared to most other phylotypes. Nontypeable strains also showed high rates of resistance. Correspondence analysis between resistance and virulence traits (Figure 4) showed that strains with more than ten virulence genes were associated with the low resistance trait, while strains classified as medium groups for virulence (five to seven genes) did not show a strong association with any resistance traits.

## 4. Discussion

*E. coli* is known to be a common cause of genitourinary infections and vulvar discharge in swine, but its full impact, particularly concerning the ExPEC strains, is not yet fully understood. Previous studies indicate that potential pathogenic *E. coli* strains in porcine and humans are classified mainly in group B2, with a smaller proportion belonging to group D. In contrast, commensal intestinal strains are predominantly classified in groups A and B1 [35,36]. Another study also found similar data in *E. coli* strains isolated from the reproductive tract of cows and sows, with the most prevalent phylotypes being A, followed by B1 [2]. Our study is in line with this report, with phylotypes B1 and A being the most prevalent. Nevertheless, we also detected the B2 and D phylotypes at a lower frequency in our study, which is consistent with the fact that the samples were collected from females with purulent vulvar discharge.

A higher proportion of strains belonging to intestinal commensal groups B1 and A is expected. Modern sows are reared in intensive systems with cages or collective pens that favor continuous contact of feces with the perineal region. Thus, it is to be expected that the vaginal canal contains most of these groups. The presence of other phylotypes considered to be of greater pathogenic risk, such as B2 and D, even if to a lesser extent, provides significant epidemiological information regarding purulent vulvar discharges. In fact, correspondence analysis in our study showed that phylotypes B2 and D were associated with a greater number of virulence factors than phylotypes A and B1.

Additionally, studies have demonstrated that commensal strains in phylotypes A and B1 exhibit a higher prevalence of drug resistance but fewer virulence genes. Conversely, pathogenic strains in phylotypes B2 and D possess several pathogenicity-associated islands and express several virulence factors [37]. This can also be seen in our resistance versus phylotype heatmap, in which it is noted that A and B1 have higher rates of resistance when compared to most other phylogroups, and to some extent, it is also seen in the second correspondence analysis, in which strains with a very high presence of virulence genes were associated with the characteristic low resistance.

The isolated *E. coli* strains showed many of the investigated extraintestinal virulence genes, which may be important in the colonization and rise of the bacteria in the genitourinary tract [38]. The association between the aerobactin operon (*iucD* and *iutA*) and the *iss* gene, both present in most strains, is related to high levels of virulence, and even the possession of any of these genes is sufficient for intermediate levels of virulence in APEC [39].

The *cvi/cva* genes and *kpsMT2*, related to the ability to overcome host defenses [26,40], were also detected in the strains. The *sat* gene was also found with relevant frequency in this study (20%). To a lesser extent, but still relevant, was the *usp* gene (9.6%). The *sat* gene is related to the secretion of a protease with cytotoxic activity in renal and bladder cells [41], while the *usp* gene (uropathogenic specific protein) is commonly found in uropathogenic strains in humans [42]. The presence of these genes indicates that these strains may have a more invasive and virulent trait.

Regarding the factors that encode important fimbriae in the adhesion and colonization of the genital and urinary epithelium, *focH*, *fimH*, and *papC* genes were found at relevant frequencies. The *papC* gene belongs to the *pap* operon that codes for the P fimbriae, which is important in urinary tract infection and potential systemic infections in poultry [43,44]. Brito et al. [9] found that up to 54.8% of *E. coli* strains isolated from pigs with urinary tract infections were *pap*-positive. Another study from Kassé et al. [45] showed that 89% and 9% of *E. coli* strains isolated from the uterus of cows with postpartum metritis possessed the genes *fimH* and *papC*, respectively, showing the potential role of these factors in the adherence of the reproductive tract by *E. coli*. Indeed, adhesion to endometrial cells is partially mediated by *fimH*, as their adhesion can be reduced by D-Mannose, an inhibitor of fimbrial adhesion [46].

Necrotizing cytotoxic factor 1 (*cnf1*) and α-hemolysin (*hlyA*) had a lower prevalence in our study. Spindola et al. [10] found a higher prevalence of this gene in UPEC strains isolated from pigs but a similar prevalence of *hlyA*.

Regarding the virulence profiles, it was observed that in the association of iron uptake genes, genes related to adhesion and attachment, serum resistance, toxin release, and bacteriocin were the most prevalent when considering the median profile. Up to 14 associated virulence genes were found (*astA*, *iss1*, *iss2*, *irp2*, *iutA*, *iucD*, *iroN*, *sfa*, *hlyF*, *papC*, *focH*, *fimH*, *cdtB*, and *csgA*). This underscores the multifaceted nature of ExPEC virulence, which may potentially facilitate the bacteria’s adaptability and persistence in the genital tract.

The tested strains of *E. coli* exhibited high resistance to several antimicrobials, including ciprofloxacin, streptomycin, cephalexin, florfenicol, tetracycline, and ampicillin. Some of these antimicrobials are commonly used in pig farming [47], including for the treatment of genitourinary infections. Similar results were found by Spindola et al. [10], who also observed high resistance of UPEC to tetracycline, florfenicol, and ampicillin, but sensitivity to ceftiofur, gentamicin, spectinomycin, and amoxicillin and clavulanic acid. In contrast, 65% of the strains in this study were resistant to amoxicillin and clavulanic acid.

Among the 135 tested strains, 128 (94.8%) were classified as multidrug-resistant (MDR), defined as resistance to at least one agent in three or more antimicrobial classes [48]. It is important to highlight that some of these antimicrobials are considered critically important in human medicine, such as cephalexin and ciprofloxacin [49]. In fact, people in the swine chain production, such as farm or slaughterhouse workers and veterinarians, are under greater exposure to pathogenic bacteria. Humans exposed to live animals were more frequently positive for zoonotic bacteria [50], as well as for bacteria that carry elements of resistance, such as β-lactam-resistant *E. coli* [51].

The presence of several tested virulence genes, some with high frequency, indicates that the strains isolated from the vaginal canal of sows possess the factors that characterize extraintestinal pathogenic *E. coli* (ExPEC) and have the potential to cause infection, particularly those belonging to phylotypes with a higher risk of pathogenicity, such as B2 and D. However, more studies are needed to elucidate the precise roles and interrelationships of these genes, as well as the relationship with other bacteria present in the genital tract that could be pivotal in the emergence of urogenital infections. This will also help in the development of specific strategies for the prevention and treatment of infections caused by virulent strains of *E. coli*. The observed high levels of resistance, with over 90% of the strains classified as multidrug-resistant, strongly indicate the indiscriminate use of antimicrobials in pigs housed on Brazilian farms.

## 5. Conclusions

*E. coli* strains isolated from the vaginal canal of sows with purulent vulvar discharge harbor a notable array of extraintestinal virulence genes that facilitate their colonization and potential infection in the genitourinary tract. The identification of genes related to survival outside the gut, associated with genes that confer serum resistance and adhesion to the genitourinary tract epithelium, indicates a propensity of these strains to circumvent host defenses, marking them as potentially more invasive and virulent.

The detection of B2 and D phylotypes, known for their pathogenic potential, adds substantial information to the epidemiology of purulent vulvar discharges and correlates with a higher number of virulence factors compared to A and B1 groups, emphasizing their potential role in infections. Furthermore, the alarming prevalence of multidrug resistance seen in most of the tested strains calls for a critical review of the antimicrobial use in pig farming, particularly to preserve the efficacy of drugs that are critically important in human medicine.

## Figures and Tables

**Table 1:** Frequency of positive strains for genes encoding virulence factors for extraintestinal infection.

Gene	Frequency (%)
*iutA*	100.0
*iucD*	92.6
*csgA*	64.4
*iss2*	58.5
*irp2*	57.8
*iroN*	54.1
*astA*	44.4
*crl*	38.5
*hlyF*	37.8
*fimH*	37.0
*cvi/cva*	28.9
*papC*	25.9
*sat*	20.0
*focH*	12.6
*kpsMTII*	9.6
*sfa*	9.6
*usp*	9.6
*iss1*	8.1
*ibeA*	3.7
*neuS*	3.7
*vat*	3.7
*cdtB*	1.5
*cnf1*	0.7
*hlyA*	0.7
*fyuA*	0.0
*afa*	0.0
*afabc*	0.0
*papE*	0.0
*kpsMTIII*	0.0

**Table 2:** Distribution of strains according to MIC values, respective MIC_50_ and MIC_90_ values, and resistance rates for assessed antimicrobials. The vertical black bars indicate the cutoff points for bacterial resistance classification.

Antimicrobial	MIC (µg/mL)																								MIC_50_ (µg/mL)	MIC_90_ (µg/mL)	Resistance (%)
	≤0.06	0.06		0.12	0.25		0.5	1		2	4		8		16	32		64	128		256	512		>512			
Ceftiofur					108		17	2		0	0		1		7										≤0.25	0.5	5.9
Ampicillin								4		17	6		4		2	1		0	101						>64	>64	75.6
Cephalexin							2	0		0	27		73		16	5		12							8	32	78.5
Oxytetracycline										27	5		0		0	0		103							>32	>32	76.3
Florfenicol							1	0		3	25		13		93										>8	>8	78.5
Marbofloxacin	4	0		0	19		21	18		7	7		59												2	>4	48.9
Enrofloxacin					16		20	20		7	7		65												4	>4	53.3
Ciprofloxacin								4		13	19		99												>4	>4	87.4
Gentamicin							86	17		4	2		5		9	12									≤0.5	16	15.6
Neomycin											119		1		4	11									≤4	16	11.9
Spectinomycin													59		48	14		6	5		3				16	64	5.9
Streptomycin								1		10	14		1		4	105									>16	>16	81.5
Sulfamethoxazole																			49		37	47		2	256	512	36.3
Azithromycin											39		33		8	9		10	36						8	>64	40.7
Colistin								58		71	3		0		0	3									2	2	4.4
Fosfomycin													119		5	0		1	2		1	1		6	≤8	16	5.9
**Antimicrobial**	**MIC (µg/mL)**																								**MIC_50_** **(µg/mL)**	**MIC_90_** **(µg/mL)**	**Resistance** **(%)**
	**≤1/0.5**		**1/0.5**			**2/1**			**4/2**			**8/4**		**16/8**			**32/16**			**64/32**			**>64/32**				
Amoxicillin/Clavulanic acid	2		0			2			12			14		20			74			8			3		32/16	32/16	63.0
	**≤1/19**		**1/19**			**2/38**			**4/76**			**>4/76**		-			-			-			-				
Trimethoprim/Sulfamethoxazole	102		0			0			0			33													≤1/9	>4/76	24.4

## Data Availability

The data presented in this study are available on request from the corresponding author.

## Supplementary Materials

The following supporting information can be downloaded at https://www.mdpi.com/article/10.3390/microorganisms12010123/s1. Table S1: Primers used to amplify *E. coli* virulence genes related to extra intestinal infections; Table S2: *p*-values for Chi-square or Fisher exact test for prevalence of virulence gene in B2 phylotype vs. other phylotypes.

## References

[1] Croxen M.A., Finlay B.B. (2010). Molecular Mechanisms of *Escherichia coli* Pathogenicity. Nat. Rev. Microbiol..

[2] Torres Luque A., Gonzalez Moreno C., Pasteris S.E., Orden J.A., de la Fuente R., Otero M.C. (2017). Antimicrobial Resistant *Escherichia coli* in the Reproductive Tract Microbiota of Cows and Sows. Comp. Immunol. Microbiol. Infect. Dis..

[3] Isling L.K., Aalbaek B., Schrøder M., Leifsson P.S. (2010). Pyelonephritis in Slaughter Pigs and Sows: Morphological Characterization and Aspects of Pathogenesis and Aetiology. Acta Vet. Scand..

[4] Kirkwood R.N., Althouse G.C., Yaeger M.J., Carr J., Almond G.W., Zimmerman J.J., Karriker L.A., Ramirez A., Schwartz K.J., Stevenson G.W. (2012). Diseases of Reproductive System. Diseases Of Swine.

[5] Poolman J.T., Wacker M. (2016). Extraintestinal Pathogenic *Escherichia coli*, a Common Human Pathogen: Challenges for Vaccine Development and Progress in the Field. J. Infect. Dis..

[6] Ding Y., Tang X., Lu P., Wu B., Xu Z., Liu W., Zhang R., Bei W., Chen H., Tan C. (2012). Clonal Analysis and Virulent Traits of Pathogenic Extraintestinal *Escherichia coli* Isolates from Swine in China. BMC Vet. Res..

[7] Krag L., Hancock V., Aalbaek B., Klemm P. (2009). Genotypic and Phenotypic Characterisation of *Escherichia coli* Strains Associated with Porcine Pyelonephritis. Vet. Microbiol..

[8] Tan C., Tang X., Zhang X., Ding Y., Zhao Z., Wu B., Cai X., Liu Z., He Q., Chen H. (2012). Serotypes and Virulence Genes of Extraintestinal Pathogenic *Escherichia coli* Isolates from Diseased Pigs in China. Vet. J..

[9] de Brito B.G., da Silva Leite D., Linhares R.E.C., Vidotto M.C. (1999). Virulence-Associated Factors of Uropathogenic *Escherichia coli* Strains Isolated from Pigs. Vet. Microbiol..

[10] Spindola M.G., Cunha M.P.V., Moreno L.Z., Amigo C.R., Silva A.P.S., Parra B.M., Poor A.P., de Oliveira C.H., Perez B.P., Knöbl T. (2018). Genetic Diversity, Virulence Genotype and Antimicrobial Resistance of Uropathogenic *Escherichia coli* (UPEC) Isolated from Sows. Vet. Q..

[11] Clermont O., Christenson J.K., Denamur E., Gordon D.M. (2013). The Clermont *Escherichia coli* Phylo-Typing Method Revisited: Improvement of Specificity and Detection of New Phylo-Groups: A new *E. Coliphylo*-Typing Method. Environ. Microbiol. Rep..

[12] Clermont O., Dixit O.V.A., Vangchhia B., Condamine B., Dion S., Bridier-Nahmias A., Denamur E., Gordon D. (2019). Characterization and Rapid Identification of Phylogroup G in *Escherichia coli*, a Lineage with High Virulence and Antibiotic Resistance Potential. Environ. Microbiol..

[13] Hijazin M., Hassan A.A., Alber J., Lämmler C., Timke M., Kostrzewa M., Prenger-Berninghoff E., Zschöck M. (2012). Evaluation of Matrix-Assisted Laser Desorption Ionization-Time of Flight Mass Spectrometry (MALDI-TOF MS) for Species Identification of Bacteria of Genera Arcanobacterium and Trueperella. Vet. Microbiol..

[14] Boom R., Sol C.J., Salimans M.M., Jansen C.L., Wertheim-van Dillen P.M., van der Noordaa J. (1990). Rapid and Simple Method for Purification of Nucleic Acids. J. Clin. Microbiol..

[15] Jansen T. (2001). Virulence-Associated Genes in Avian Pathogenic (APEC) Isolated from Internal Organs of Poultry Having Died from Colibacillosis. Int. J. Med. Microbiol..

[16] Yamamoto S., Terai A., Yuri K., Kurazono H., Takeda Y., Yoshida O. (1995). Detection of Urovirulence Factors in *Escherichia coli* by Multiplex Polymerase Chain Reaction. FEMS Immunol. Med. Microbiol..

[17] Ewers C., Li G., Wilking H., Kiessling S., Alt K., Antáo E.-M., Laturnus C., Diehl I., Glodde S., Homeier T. (2007). Avian Pathogenic, Uropathogenic, and Newborn Meningitis-Causing *Escherichia coli*: How Closely Related Are They?. Int. J. Med. Microbiol..

[18] Ewers C., Janssen T., Kiessling S., Philipp H.-C., Wieler L.H. (2004). Molecular Epidemiology of Avian Pathogenic *Escherichia coli* (APEC) Isolated from Colisepticemia in Poultry. Vet. Microbiol..

[19] Horne S.M., Pfaff-McDonough S.J., Giddings C.W., Nolan L.K. (2000). Cloning and Sequencing of the Iss Gene from a Virulent Avian *Escherichia coli*. Avian Dis..

[20] Johnson T.J., Wannemuehler Y.M., Nolan L.K. (2008). Evolution of the Iss Gene in *Escherichia coli*. Appl. Environ. Microbiol..

[21] Schubert S., Rakin A., Karch H., Carniel E., Heesemann J. (1998). Prevalence of the “High-Pathogenicity Island” of Yersinia Species among *Escherichia coli* Strains That Are Pathogenic to Humans. Infect. Immun..

[22] Bauer R.J., Zhang L., Foxman B., Siitonen A., Jantunen M.E., Saxen H., Marrs C.F. (2002). Molecular Epidemiology of 3 Putative Virulence Genes for *Escherichia coli* Urinary Tract Infection-Usp, Iha, and iroN (*E. Coli*). J. Infect. Dis..

[23] Tsukamoto T. (1997). PCR method for detection of K1 antigen and serotypes of Escherichia coli isolated from extraintestinal infection. Kansenshogaku Zasshi.

[24] Yamamoto T., Echeverria P. (1996). Detection of the Enteroaggregative *Escherichia coli* Heat-Stable Enterotoxin 1 Gene Sequences in Enterotoxigenic *E. coli* Strains Pathogenic for Humans. Infect. Immun..

[25] Johnson J.R., Stell A.L. (2000). Extended Virulence Genotypes of *Escherichia coli* Strains from Patients with Urosepsis in Relation to Phylogeny and Host Compromise. J. Infect. Dis..

[26] Maurer J.J., Brown T.P., Steffens W.L., Thayer S.G. (1998). The Occurrence of Ambient Temperature-Regulated Adhesins, Curli, and the Temperature-Sensitive Hemagglutinin Tsh among Avian *Escherichia coli*. Avian Dis..

[27] Johnson T.J., Wannemuehler Y., Doetkott C., Johnson S.J., Rosenberger S.C., Nolan L.K. (2008). Identification of Minimal Predictors of Avian Pathogenic *Escherichia coli* Virulence for Use as a Rapid Diagnostic Tool. J. Clin. Microbiol..

[28] (2019). Performance Standards for Antimicrobial Disk and Dilution Susceptibility Tests for Bacteria Isolated From Animals.

[29] Schwarz S., Silley P., Simjee S., Woodford N., van Duijkeren E., Johnson A.P., Gaastra W. (2010). Editorial: Assessing the Antimicrobial Susceptibility of Bacteria Obtained from Animals. J. Antimicrob. Chemother..

[30] R Core Team (2023). R: A Language and Environment for Statistical Computing.

[31] Gu Z., Eils R., Schlesner M. (2016). Complex Heatmaps Reveal Patterns and Correlations in Multidimensional Genomic Data. Bioinformatics.

[32] Kassambara A., Mundt F. (2020). Factoextra. http://www.sthda.com/english/rpkgs/factoextra.

[33] Lê S., Josse J., Husson F. (2008). FactoMineR: An R Package for Multivariate Analysis. J. Stat. Softw..

[34] Wickham H. (2016). Ggplot2: Elegant Graphics for Data Analysis.

[35] Bok E., Kożańska A., Mazurek-Popczyk J., Wojciech M., Baldy-Chudzik K. (2020). Extended Phylogeny and Extraintestinal Virulence Potential of Commensal *Escherichia coli* from Piglets and Sows. Int. J. Environ. Res. Public Health.

[36] Picard B., Garcia J.S., Gouriou S., Duriez P., Brahimi N., Bingen E., Elion J., Denamur E. (1999). The Link between Phylogeny and Virulence in *Escherichia coli* Extraintestinal Infection. Infect. Immun..

[37] Smith J.L., Fratamico P.M., Gunther N.W. (2007). Extraintestinal Pathogenic *Escherichia coli*. Foodborne Pathog. Dis..

[38] Wiles T.J., Kulesus R.R., Mulvey M.A. (2008). Origins and Virulence Mechanisms of Uropathogenic *Escherichia coli*. Exp. Mol. Pathol..

[39] Tivendale K.A., Allen J.L., Ginns C.A., Crabb B.S., Browning G.F. (2004). Association of Iss and iucA, but Not Tsh, with Plasmid-Mediated Virulence of Avian Pathogenic *Escherichia coli*. Infect. Immun..

[40] Sirlici M.P., Trabulsi L.R., Trabulsi L.R., Alterthum F. (2005). Fatores de Virulência: Adesão, Invasão, Sideróforos, Evasinas. Microbiologia.

[41] Guyer D.M., Henderson I.R., Nataro J.P., Mobley H.L. (2000). Identification of Sat, an Autotransporter Toxin Produced by Uropathogenic *Escherichia coli*. Mol. Microbiol..

[42] Nipič D., Podlesek Z., Budič M., Črnigoj M., Žgur-Bertok D. (2013). *Escherichia coli* Uropathogenic-Specific Protein, Usp, Is a Bacteriocin-like Genotoxin. J. Infect. Dis..

[43] Dho-Moulin M., Fairbrother J.M. (1999). Avian Pathogenic *Escherichia coli* (APEC). Vet. Res..

[44] Kariyawasam S., Johnson T.J., Nolan L.K. (2006). The Pap Operon of Avian Pathogenic *Escherichia coli* Strain O1:K1 Is Located on a Novel Pathogenicity Island. Infect. Immun..

[45] Kassé F.N., Fairbrother J.M., Dubuc J. (2016). Relationship between *Escherichia coli* Virulence Factors and Postpartum Metritis in Dairy Cows. J. Dairy Sci..

[46] Sheldon I.M., Rycroft A.N., Dogan B., Craven M., Bromfield J.J., Chandler A., Roberts M.H., Price S.B., Gilbert R.O., Simpson K.W. (2010). Specific Strains of *Escherichia coli* Are Pathogenic for the Endometrium of Cattle and Cause Pelvic Inflammatory Disease in Cattle and Mice. PLoS ONE.

[47] Dutra M.C., Moreno L.Z., Dias R.A., Moreno A.M. (2021). Antimicrobial Use in Brazilian Swine Herds: Assessment of Use and Reduction Examples. Microorganisms.

[48] Magiorakos A.-P., Srinivasan A., Carey R.B., Carmeli Y., Falagas M.E., Giske C.G., Harbarth S., Hindler J.F., Kahlmeter G., Olsson-Liljequist B. (2012). Multidrug-Resistant, Extensively Drug-Resistant and Pandrug-Resistant Bacteria: An International Expert Proposal for Interim Standard Definitions for Acquired Resistance. Clin. Microbiol. Infect..

[49] WHO (World Health Organization) (2018). WHO Advisory Group on Integrated Surveillance of Antimicrobial Resistance (AGISAR)—Critically Important Antimicrobials for Human Medicine.

[50] Klous G., Huss A., Heederik D.J.J., Coutinho R.A. (2016). Human–Livestock Contacts and Their Relationship to Transmission of Zoonotic Pathogens, a Systematic Review of Literature. One Health.

[51] Dang S.T.T., Bortolaia V., Tran N.T., Le H.Q., Dalsgaard A. (2018). Cephalosporin-resistant *Escherichia coli* Isolated from Farm Workers and Pigs in Northern Vietnam. Trop. Med. Int. Health.

