# Supplementary material for: Characterization of Escherichia coli Isolated from Sows Presenting Purulent Vulvar Discharge
Source: Microorganisms. 2024 Jan 8;12(1):123. doi: 10.3390/microorganisms12010123 (PMC10820650; doi:10.3390/microorganisms12010123)
Supplement: Supplementary file 1 [file microorganisms-12-00123-s001.zip › microorganisms-2794330-supplementary.pdf]

**Table S1:** Primers used to amplify *E. coli* virulence genes related to extra intestinal infections.

| Gene | Primer sequence (5'-3') | Amplicon (bp) | Reference |
|---|---|---|---|
| *papC* | TGATATCACGCAGTCAGTAGC<br>CCGGCCATATTCACATAA | 501 | [12] |
| *hlyA* | AACAAGGATAAGCACTGTTCTGGCT<br>ACCATATAAGCGGTCATTCCCGTCA | 1177 | [13] |
| *cnf1* | AAGATGGAGTTTCCTATGCAGGAG<br>CATTCAGAGTCCTGCCCTCATTATT | 498 | [13] |
| *Sat* | TGCTGGCTCTGGAGGAAC<br>TTGAACATTCAGAGTACCGGG | 667 | [14] |
| *vat* | TCCTGGGACATAATGGTCAG<br>GTGTCAGAACGGAATTG | 981 | [15] |
| *iucD* | TACCGGATTGTCATATGCAGACCGT<br>AATATCTTCCTCCAGTCCGGAGAAG | 602 | [13] |
| *iss1* | GTGGCGAAAACTAGTAAAACAGC<br>CGCCTCGGGGTGGATAA | 760 | [16] |
| *iss2* | CAGCAACCCGAACCACTTGATG<br>AGCATTGCCAGAGCGGCAGAA | 323 | [17] |
| *lrp2* | AAGGATTCGCTGTTACCGGAC<br>TCGTCGGGCAGCGTTTCTTCT | 281 | [18] |
| *fyuA* | GCGAC GGGAAGCGA TTTA<br>CGCAGTAGGCACGATGTTGTA | 780 | [18] |
| *usp* | ACATTCACGGCAAGCCTCAG<br>AGCGAGTTCCTGGTGAAAGC | 440 | [19] |
| *neuS* | TATAATTAGTAACCTGGGGC<br>GGCGCTATTGAATAAGACTG | 927 | [20] |
| *cvi/cva* | TCCAAGCGGACCCCTTATAG<br>CGCAGCATAGTTCCATGCT | 598 | [14] |
| *astA* | TGCCATCAACACAGTATATCC<br>TAGGATCCTCAGGTCGCGAGTGACGGC | 116 | [21] |
| *iutA* | GGCTGGACATCATGGGAACTGG<br>CGTCGGGAACGGGTAGAATCG | 302 | [22] |
| *kpsMT-II* | GCGCATTTGCTGATACTGTTG<br>CATCCAGACGATAAGCATGAGCA | 272 | [22] |
| *kpsMT-III* | TCCTCTTGCTATTATTCCCCCT<br>AGGCGTATCCATCCCTCCTAAC | 392 | [22] |
| *crl* | TTTCGATTGTCTGGCTGTATG<br>CTTCAGATTCAGCGTCGTC | 250 | [23] |
| *csgA* | ACTCTGACTTGACTATTACC<br>AGATGCAGTCTGGTCAAC | 200 | [22] |
| *afaBC* | GCTGGGCAGCAAACTGATAACCTC<br>CATCAAGCTGTTTGTTCGTCCGCCG | 750 | [13] |

| | | | |
|---|---|---|---|
| *hlyF* | GGCCACAGTCGTTTAGGGTGCTTACC<br>GGCGGTTTAGGCATTCCGATACTCAG | 450 | [24] |
| *iroN* | AATCCGGCAAAGAGACGAACCGCCT<br>GTTCGGGCAACCCCTGCTTTGACTTT | 553 | [24] |
| *ibeA* | TGGAACCCGCTCGTAATATAC<br>CTGCCTGTTCAAGCATTGCA | 342 | [15] |
| *sfa* | CTCCGGAGAACTGGGTGCATCTTAC<br>CGGAGGAGTAATTACAAACCTGGCA | 410 | [22] |
| *focH* | ATGCGTAAATATTATCCCCTC<br>GCCACCTGTCTGGATATAGAC | 603 | [7] |
| *fimH* | TGCAGAACGGATAAGCCGTGG<br>GCAGTCACCTGCCCTCCGGTA | 508 | [22] |
| *cdtB* | AAATCACCAAGAATCATCCAGTTA<br>AAATCTCCTGCAATCATCCAGTTTA | 430 | [22] |
| *afa* | GGCAGAGGGCCGGCAACAGGC<br>CCCGTAACGCGCCAGCATCTC | 559 | [22] |
| *papE* | GCAACAGCAACGCTGGTTGCATCAT<br>AGAGAGAGCCACTCTTATACGGACA | 336 | [22] |

**Table S2:** P-values for Chi-square or Fisher exact test for prevalence of virulence gene in B2 phylotype vs. other phylotypes.

| Gene | B2 vs B1 | B2 vs A | B2 vs C | B2 vs D | B2 vs E | B2 vs F | Unk |
|---|---|---|---|---|---|---|---|
| *papC* | 0,0587 | 0,0008 | 0,2215 | 0,4216 | 1 | 1 | 0,0128 |
| *focH* | 0,0031 | 0,012 | 0,0075 | 0,1892 | 0,6329 | 0,4697 | 0,1516 |
| *usp* | 0,00046 | 0,00013 | 0,0021 | 0,0053 | 0,1451 | 0,4545 | 0,0656 |
| *kpsMT2* | 0,00046 | 0,00013 | 0,0021 | 0,0053 | 0,0338 | 1 | 0,0656 |
| *sfa* | 0,0031 | 0,00078 | 0,0075 | 0,1892 | 0,3069 | 0,4697 | 0,0359 |
| *vat* | 0,00288 | 0,01833 | 0,0678 | 0,0462 | 0,25 | 1 | 0,5882 |
| *ibeA* | 0,0105 | 0,01833 | 0,0678 | 0,0462 | 0,25 | 1 | 0,5882 |
| *neuS* | 0,0612 | 0,07563 | 0,1779 | 0,1385 | 0,5 | 0,4545 | 1 |
| *cnf1* | 0,1563 | 0,28571 | 0,4348 | 0,3846 | 1 | 1 | 1 |
